# The Relationship between Aeroallergen Sensitization and Chronic Cough in School-Aged Children from General Population

**DOI:** 10.1155/2021/5513611

**Published:** 2021-11-18

**Authors:** Myongsoon Sung, Dong Keon Yon, Seung Won Lee, Ju Hee Kim, Hey Sung Baek, Hye Mi Jee, Youn Ho Shin, Man Yong Han

**Affiliations:** ^1^Department of Pediatrics, Soonchunhyang University Gumi Hospital, Gumi, Republic of Korea; ^2^Department of Pediatrics, Seoul National University Hospital, Seoul National University College of Medicine, Seoul, Republic of Korea; ^3^Department of Data Science, College of Software Convegence, Sejong University, Seoul, Republic of Korea; ^4^Department of Pediatrics, Hallym University Kangdong Sacred Heart Hospital, Seoul, Republic of Korea; ^5^Departments of Pediatrics, CHA Bundang Medical Center, CHA University School of Medicine, Seongnam, Republic of Korea; ^6^Department of Pediatrics, CHA Gangnam Medical Center, CHA University School of Medicine, Seoul, Republic of Korea

## Abstract

**Objective:**

Determining sensitivity to allergens is an essential step in diagnosing children with allergic diseases. Chronic cough has remained poorly understood with causative triggers. The purpose of our study was to shed light on the relationship between sensitization to aeroallergens and chronic cough.

**Methods:**

This population-based study examined children (aged 7 years to 13 years) between June and July 2016. The 1,259 children, 72 of whom (5.7%) had a chronic cough, and 1,187 of whom (94.3%) did not (controls), completed the questionnaire, but 1,051 children completed skin prick tests (SPTs) with eight aeroallergens.

**Results:**

There were positive SPT results to at least 1 allergen in 549 children (52.2%). Sensitization to house dust mite (HDM) was most common (chronic cough = 46.9%; controls = 47.2%), followed by pollen (chronic cough = 21.9%; controls = 16.5%) in both groups, but there was no difference in allergic profile and sensitization to aeroallergen (*P* > 0.05 for all comparisons). Multivariable analysis with adjustment for confounding indicated that children who were in sensitization to pollen had an increased risk of chronic cough (aOR = 2.387; 95% CI: 1.115 to 5.111; *P* = 0.025). Multivariable analysis with adjustment for confounding indicated that children who were exposed to current smoking (aOR = 4.442; 95% CI: 1.831 to 10.776; *P* = 0.001) and mold (aOR = 1.988; 95% CI: 1.168 to 3.383; *P* = 0.011) were associated with chronic cough.

**Conclusion:**

Sensitization to pollen should be considered as a potential contributing factor to the development of chronic cough in school-aged children.

## 1. Introduction

Determining sensitivity to allergens is essential in diagnosing and predicting children with allergic diseases [1]. According to allergen type, sensitization to allergens could be different during a lifetime [2]. Nowadays, sensitization to pollen is increasing in children globally, and pollen allergy is strongly associated with a significant number of hospital visits worldwide [3, 4].

Meanwhile, chronic cough is defined as a cough that lasts more than four weeks in children [5]. Because chronic cough may be the sole presenting symptom of an underlying chronic respiratory illness in children, it is common reason parents visit respiratory specialists for evaluation for their children [5–8]. Previous researches suggest that chronic cough in children may often be from allergic diseases, such as asthma and allergic rhinitis (AR) [7, 8], and almost 70% of children with a chronic cough had used asthma medications [9, 10]. Therefore, children with chronic cough may inappropriately use asthma medications if there is a misdiagnosis. Despite the significance of chronic cough as a possible indicator of allergic disease or chronic lung disease in children, most studies of children examined those who were under four years old or attended child care centers [6–8], not school-aged children. Therefore, chronic cough risk factors and etiology in school-aged children remain poorly understood with causative triggers. From these results, we postulate that sensitization to pollen may be related to chronic cough in children.

However, to our knowledge, no comprehensive studies to date have assessed a relationship between sensitization to aeroallergen and chronic cough in school-aged children from the general population. Therefore, the purpose of our study was to shed light on the relationship between sensitization to aeroallergens including pollen, house-dust mite (HDM), and animal dander and chronic cough to use skin prick tests (SPTs) and questionnaires. Also, we identify the relationships to chronic cough with the demographic or environmental characteristics, including gender, allergic comorbidities, family history, and exposure to tobacco smoke and mold, and estimate the prevalence of chronic cough in school-aged children.

## 2. Materials and Methods

### 2.1. Study Design

This prospective cross-sectional study examined 1,259 Korean children from the general population who were 7 to 13 years old and attended 6 different elementary schools between June and July 2016. The parents of each included child completed a chronic cough questionnaire (with questions about the characteristics, duration, and triggering factors) and an environment questionnaire, before the physical examination and SPT. The students underwent the physical examinations and SPT in their respective schools by trained field technicians. Among the 1,259 children whose parents returned completed questionnaires, 208 children were excluded because they had unusable SPT results.

### 2.2. Diagnosis of Allergic Diseases

The diagnosis of allergic diseases (asthma, AR, and atopic dermatitis (AD)) was based on the Korean International Study of Asthma and Allergies in Childhood (ISAAC) questionnaire, according to the past 12-month history of each child [11, 12]. This questionnaire provided data on the general characteristics of each child, including gender, birth date, prenatal and postnatal history, height, weight, and parental history of allergic diseases (asthma, AR, and AD).

### 2.3. Measurement of Atopy

An SPT was used to measure atopy using standardized allergen extracts and control solutions from LaForma (Milan, Italy) on the volar surface of both arms and on the skin that had normal appearance. Subjects were tested for sensitivity to the following eight common aeroallergens: HDM (*Dermatophagoides pteronyssinus* and *Dermatophagoides farinae*), animal dander (cat and dog), Alternaria, and pollen (birch, oak, and Japanese hop). A positive SPT result was defined by a mean wheal diameter of 3 mm or more than the positive control. Nonsensitization was defined as having no positive allergenic reactions. Allergic sensitization was defined by the presence of one or more positive reactions in the SPT [12], which are monosensitization as having positive reaction to a single antigen or multiple cross-reactive antigens without other positive tests and polysensitization as positive reactions to antigens in different classes. Sensitization to pollen was defined having positive sensitization to elm, birch, oak, and Japanese hop.

### 2.4. Definition and Questionnaire of Chronic Cough

Based on previous studies [5, 6], a “chronic cough” was defined by an affirmative answer to the question: “In the past 12 months, has your child had a problem with a cough whose duration was more than 4 weeks?” The period of the chronic cough was also recorded (4 weeks ago vs. more than 4 weeks ago). The particular characteristics of the cough were also recorded, including “hoarseness,” “yellowish rhinorrhea,” “nasal stuffiness or postnasal dripping,” “sputum or neck trimming,” “aggravation or disappearance of coughing during sleep,” “association with eating food,” “chest pain,” “dyspnea,” and “hemoptysis.”

Among the many questionnaire items, we further analyzed specific items that had significant associations with chronic cough in previous studies [5–10]. Pet owners were defined by an affirmative response to the question, “Do you have any pets in your household living with your child?”; the parents were then asked to report the type of animal(s). The presence of “visual mold” was defined by an affirmative answer to the question, “Do you see any wet moldy spots on the ceilings or walls in your household?” and “smelling mold” as an affirmative answer to the question, “Do you smell mold in your household?” The constitutional and environmental factors examined were number of family members living together; presence, number, and birth order of other siblings; type of delivery (vaginal vs. C-section); birth weight; exposure to current secondary tobacco smoke (yes vs. no); duration of the residence (less than 6 years vs. 6 years or more); presence of mold on the walls or ceiling of the residence; type of ventilation; change of residence since birth; and remodeling of the residence (replacing wallpaper, flooring, ceiling, etc.).

### 2.5. Ethics

The study protocol was approved by the Institutional Review Board of the CHA Bundang Medical Center (IRB No. 2016-04-032). Written informed consent was obtained from the parents or guardians of all participants following a detailed explanation of the study.

### 2.6. Statistical Analysis

All statistical analyses were performed using IBM SPSS Statistics 23.0 (IBM Corp., Armonk, NY, USA). Each value is reported as median (interquartile range (IQR)), unless otherwise stated. Frequencies and continuous variables were compared using the *χ*^2^ test and Student's *t*-test, respectively. Statistical significance was defined as a *P* value below 0.05. We first identified demographic factors that affected chronic cough using logistic regression. Then, we identified the independent association factors of chronic cough using multivariate logistic regression, after adjustment for confounding by gender, grade in school (first/second vs. third/fourth vs. fifth/sixth), obesity (body mass index (BMI) *z*score > 1.63), sensitization to aeroallergen (no vs. yes), premature birth (gestational age < 37 weeks), and/or low birth weight. To analyze environmental association factors, we corrected for confounding by factors that affected chronic cough (gender, school grade, sensitization to pollen, and current asthma).

## 3. Results

### 3.1. General Characteristics

Table 1 shows the characteristics of 1,259 children, 72 of whom (5.7%) had a chronic cough, and 1,187 of whom (94.3%) did not (controls). Children with chronic cough were younger (*P* = 0.021) and in lower school grades (*P* = 0.030). Children with chronic cough were more likely to have parents with AR (*P* = 0.030) and AD (*P* = 0.008), and more likely to asthma (*P* < 0.001) and AR (*P* = 0.010). However, the two groups had no significant differences in sex, BMI, obesity, prenatal history (vaginal delivery, prematurity, and low birth weight), and duration of residence (Table 1). Data on the history of current smoking exposure were available for 1,255 children. A total of 46 children were exposed to tobacco smoke, 7 in the chronic cough group and 39 in the control group. Meanwhile, data on the history of mold exposure were available for 1,256 children. A total of 293 children were exposed to mold, 25 in the chronic cough group and 268 in the control group. There were differences in exposure to current smoking (*P* = 0.005) and mold (*P* = 0.019) between the two groups (Table 1).

### 3.2. The Characteristics of Children with Chronic Cough

**Figure 1:**
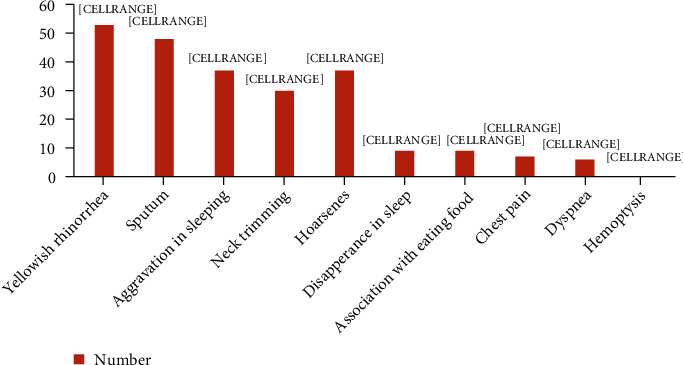
The number of characteristics of the cough in school children with chronic coughing (*n* = 72).

Figure 1 shows the characteristics of 72 children with chronic cough. The symptoms of children with chronic cough were yellowish rhinorrhea (*n* = 53, 73.6%), sputum (*n* = 48, 66.7%), aggravation while sleeping (*n* = 37, 51.3%), neck trimming (*n* = 30, 41.6%), hoarseness (*n* = 37, 51.3%), disappearance during sleep (*n* = 9, 12.5%), association with eating food (*n* = 9, 12.5%), chest pain (*n* = 7, 9.7%), and hemoptysis (*n* = 0).

### 3.3. Comparison between Chronic Cough and Control Groups in Allergen Sensitization

The 1,051 children from 64 (chronic cough) and 987 (control) children had appropriate SPTs for the final analysis because 208 children (16.5%) had unusable SPT results (Table 2). Significantly, the two groups had no difference in allergic profile and sensitization to aeroallergen (*P* > 0.05 for all comparisons). There were positive SPT results to at least 1 allergen in 549 children among the 1,051 children. The 549 children included 31 of 64 children (48.4%) with chronic cough and 518 of 987 children (52.5%) without chronic cough. The 329 children from 313 (control, 31.7%) and 16 (chronic cough, 25.0%) children were monosensitized, and 220 children from 205 (control, 20.8%) and 15 (chronic cough, 23.4%) children were polysensitized. Sensitization to HDM was the most common (chronic cough: *n* = 30, 46.9%; controls: *n* = 466, 47.2%), followed by pollen (chronic cough: *n* = 14, 21.9%; controls: *n* = 163, 16.5%) in both groups. The 26 children from 25 (control, 2.5%) and 1 (chronic cough, 1.6%) children had sensitization to mold (Table 2).

### 3.4. Associations of Chronic Coughing with Sensitization to Aeroallergen

Multivariable analysis with adjustment for confounding (gender, grade, obesity, sensitization, prematurity, and/or low birth weight) indicated that children who were in sensitization to pollen had an increased risk of chronic cough (adjusted odds ratio (aOR) = 2.387; 95% confidence interval (CI): 1.115 to 5.111; *P* = 0.025). However, sensitization to aeroallergen, HDM, and animal hair was not significantly associated with chronic cough (*P* > 0.05 for all comparisons) (Table 2).

### 3.5. Associations of Chronic Cough with Constitutional Factors

Multivariable analysis with adjustment for confounding (gender, school grade, obesity, sensitization to aeroallergen, prematurity, and/or low birth weight) indicated that children who were in fifth and sixth grade (aOR = 0.327; 95% CI: 0.126 to 0.849; *P* = 0.022) were significantly associated with chronic cough, and there was a significant association between chronic cough in children and asthma (aOR = 4.116; 95% CI: 1.913 to 8.857; *P* < 0.001). However, children with male gender, obesity, pet owner, remodeling history, AR, and AD were not significantly associated with chronic cough (*P* > 0.05 for all comparisons) (Table 3).

### 3.6. Associations of Chronic Cough with Environmental Factors

Concerning environmental factors, multivariable analysis with adjustment for confounding (gender, school grade, sensitization to pollen, and current asthma) indicated that children who were exposed to current smoking (aOR = 4.442; 95% CI: 1.831 to 10.776; *P* = 0.001) and mold (aOR = 1.988; 95% CI: 1.168 to 3.383; *P* = 0.011) were significantly associated with a chronic cough in school-aged children (Table 3).

## 4. Discussion

The present study was a cross-sectional study that used a questionnaire and SPT results to investigate the relationship between sensitization to aeroallergen and chronic cough in the general Korean population of school-aged children. We found that after adjustment for confounding factors, sensitization to pollen and allergy disease such as asthma and AR were associated with a chronic cough in school-aged children. The result agrees with previous studies [8, 13, 14] that chronic cough is closely related to allergy. These associations may be because several pathological conditions of the sensory nerve endings that regulate the cough reflex are affected by rhinitis, rhinosinusitis, and asthma [14].

Determining sensitivity to allergens is essential for the diagnosis and management of a patient with allergic diseases. Also, one study suggested that patients with AR or a history of angioedema should educate on the risks of allergen sensitization [15]. We initially hypothesized a positive association of chronic cough with sensitization to HDM and polysensitization, but our results indicated no association. Because of climate change, the sensitization to pollen has increased in the world [16]. Additionally, the sensitization rate to pollen has been one of major aeroallergen to children with allergic disease such as asthma and AR [3, 4]. The allergen sensitization rate to pollen was 16.5% (control) and 21.9% (chronic cough), which was the second-highest among examined allergens in the present study, lower than reported in the previous study from school-aged children with AR [17]. It is well-known that pollen and HDM allergens induce Th2 immune responses, but pollen grains contain other immunomodulatory substances that differentiate them from HDM [18]. Also, pollen-derived nonallergenic substances might contribute to B cell-dependent activation of IgE-mediated allergy [18]. Therefore, our finding of a difference between pollen and HDM sensitization and chronic cough may be explained by the different immunopathophysiological responses to these aeroallergens. Additionally, this study was conducted from June to July, and studies conducted during other months could produce different results.

Interestingly, we found that exposure to current smoke and mold is strongly associated with a chronic cough in school-aged children. Previous studies of children with cough, irrespective of the cause, reported that those exposed to tobacco smoke had an increased risk of chronic or recurrent cough [19, 20] in agreement with the present study. Cigarette smoke contains many different chemicals and is the major risk factor for chronic cough in adults. In particular, the prevalence of chronic cough in adults was reported as 3% in never smokers, 4% in former smokers, and 8% in current smokers [20]. Additionally, exposure to second-hand smoke leads to coughing and other respiratory symptoms [21, 22]. Our findings that exposure to tobacco smoke increased the risk of chronic cough in children are consistent with these previous results [21, 22].

Previous research showed that mold exposure is strongly related to severe respiratory symptoms of asthma, and sensitization to mold is a risk factor for life-threatening conditions in children with asthma [23]. However, our findings had a limitation by the presence of recall bias by the parents or by the very small number of children who were sensitized to mold (1 of 72 chronic coughs and 25 of 1,198 controls). Additionally, we found no significant association between gender, obesity, prenatal history, pet, remodeling history, duration of the residence, and chronic cough.

The prevalence of chronic cough in children depends on many factors, including the definition of chronicity, age, and other demographic and clinical characteristics [5]. Therefore, it is difficult to estimate accurately. We found that the overall prevalence of chronic cough was 5.7% in our school-aged children. This prevalence is similar to a previous study that reported a prevalence of 5 to 10% in primary school children (6–12 years) [24], but lower than that previously reported for children aged 8 to 11 years (about 10%) [25]. The prevalence of chronic cough is likely higher in preschool children, and retrospective [5] and prospective studies have shown that the median age of children with chronic cough seen in clinics was 2 to 3 years [26, 27]. This is in agreement with our study, which found that the prevalence of chronic cough decreased with age and school grade.

However, the current study had a few limitations. All of the 1,259 children lived in one urban area in Korea, so this study could be subject to selection bias and may have limited generalizability. This study was a cross-sectional analysis using questionnaires; thus, there is the possibility of recall bias by the parents. Nonetheless, the present study had numerous strengths. We had a large sample size, used a comprehensive and stringent questionnaire, and used SPT results, which together provided accurate and objective data. Another strength is that we examined the effect of various factors on chronic cough, such as mold, pets, and duration of the residence.

## 5. Conclusions

Overall, our study of school-aged children indicated that sensitization to pollen and exposure to tobacco smoke and mold increased the risk of chronic cough. Therefore, strategies to prevent and treat chronic cough in school-aged children should focus on these modifiable risk factors. Our results suggest that sensitization to pollen should be considered as a potential contributing factor to the development of chronic cough in school-aged children.

## Figures and Tables

**Table 1:** Demographic and clinical characteristics of the total study population (*n* = 1,259).

Characteristics	Control (*n* = 1,187)		Chronic cough (*n* = 72)		*P* value^∗∗^
Demographics	*n* ^∗^ (%)	Mean (SD) or *n* (%)	*n* ^∗^ (%)	Mean (SD) or *n* (%)	
Gender, male	1,183 (100)	607 (51.3)	72 (100)	40 (55.6)	0.484
Age, years		8.88 (1.79)		8.43 (1.55)	**0.021**
Grade	1,186 (100)		72 (100)		**0.030**
Low (1^st^-2^nd^)		618 (52.1)		45 (62.5)	
Middle (3^rd^-4^th^)		295 (24.9)		20 (27.8)	
High (5^th^-6^th^)		273 (23,0)		7 (9.7)	
Anthropometrics	1,085 (100)		66 (100)		
BMI *z* score, mean (SD)		-0.10 (1.06)		-0.14 (1.18)	0.773
Height, cm, mean (SD)		132.6 (12.9)		129.5 (12.2)	0.059
Obesity^∗∗∗^		57 (5.3)		5 (7.6)	0.417
Prenatal history^∗^					
Vaginal delivery	1,182 (100)	809 (68.4)	72 (100)	50 (69.4)	0.859
Prematurity	1,092 (100)	109 (10.0)	70 (100)	9 (12.9)	0.440
Low birth weight (<2.5 kg)	1,168 (100)	54 (4.6)	72 (100)	5 (6.9)	0.369
Duration of residence	1,187 (100)		72 (100)		0.084
>6 years		363 (30.6)		29 (40.3)	
≤6 years		824 (69.4)		42 (58.3)	
Exposure to current smoking	1,183 (100)		72 (100)		**0.005**
No		1,144 (96.7)		65 (90.3)	
Yes		39 (3.3)		7 (9.7)	
Exposure to mold	1184 (100)		72 (100)		**0.019**
No		916 (77.4)		47 (65.3)	
Yes		268 (22.6)		25 (34.7)	
Family allergic history^∗^					
Asthma	1,013 (100)	84 (8.3)	56 (100)	6 (10.7)	0.525
Allergic rhinitis	1,098 (100)	434 (39.5)	66 (100)	35 (53.0)	**0.030**
Atopic dermatitis	1,046 (100)	155 (14.8)	62 (100)	17 (27.4)	**0.008**
Allergic history^∗^					
Asthma	1,177 (100)	51 (4.3)	71 (100)	15 (22.7)	**<0.001**
Allergic rhinitis	1,187 (100)	669 (56.4)	71 (100)	51 (71.8)	**0.010**
Atopic dermatitis	1,174 (100)	252 (21.5)	72 (100)	22 (30.6)	0.071

BMI: body mass index; SD: standard deviation. ^∗^Number of completed questionnaires or of appropriate responses to questions in the questionnaire. ^∗∗^*P* values are from a *χ*^2^ test or Student's *t*-test. ^∗∗∗^Obesity was defined as BMI *z*score > 1.63. Prematurity was defined as the gestational age < 37 weeks, and low birth weight was defined as less than 2.5 kg. Numbers in bold indicate significant differences (*P* < 0.05).

**(a):** 

	Control	Chronic cough	*P* value^∗^
Allergic profiles, *n* (%)	987 (100)	64 (100)	0.529
Nonsensitization	469 (47.5)	33 (51.6)	
Sensitization	518 (52.5)	31 (48.4)	
Mono-	313 (31.7)	16 (25.0)	
Poly-	205 (20.8)	15 (23.4)	
Sensitization to aeroallergen, *n* (%)	987 (100)	64 (100)	
HDM	466 (47.2)	30 (46.9)	0.780
Animal hair	117 (11.9)	9 (14.1)	0.661
Pollen	163 (16.5)	14 (21.9)	0.361
Mold	25 (2.5)	1 (1.6)	0.497

**(b):** 

	Chronic coughing			
	Crude OR (95% CI)	*P* value	aOR (95% CI)^∗∗^	*P* value
Sensitization to aeroallergen				
No	Ref		Ref	
Yes	0.851 (0.513 to 1.411)	0.531	0.942 (0.550 to 1.613)	0.827
Sensitization to HDM				
No	Ref		Ref	
Yes	1.099 (0.619 to 1.951)	0.747	1.240 (0.673-2.286)	0.490
Sensitization to pollen				
No	Ref		Ref	
Yes	2.008 (0.967 to 4.166)	0.061	**2.387 (1.115 to 5.111)**	**0.025**
Sensitization to animal hair				
No	Ref		Ref	
Yes	1.336 (0.608 to 2.935)	0.470	1.312 (0.584 to 2.945)	0.511

aOR: adjusted odds ratio; CI: confidence interval; LBW: low birth weight; HDM: house dust mite. ^∗^*P* values are from a *χ*^2^ test or Student's *t*-test. ^∗∗^Adjusted for gender, grade, obesity, sensitization, prematurity, and/or low birth weight. Numbers in bold indicate significant differences (*P* < 0.05).

**(a):** 

Constitutional factors	Chronic coughing				
	Crude OR (95% CI)	*P* value	aOR (95% CI)^∗^	*P* value
Gender				
Girl	Ref		Ref	
Boy	1.186 (0.735 to 1.914)	0.484	1.159 (0.677 to 1.985)	0.590
Grade of school				
Low	Ref		Ref	
Medium	0.931 (0.540 to 1.605)	0.797	1.124 (0.626 to 2.018)	0.695
High	**0.352 (0.157 to 0.791)**	**0.011**	**0.327 (0.126 to 0.849)**	**0.022**
Obesity				
No	Ref		Ref	
Yes	1.478 (0.572 to 3.822)	0.420	1.700 (0.645 to 4.480)	0.827
Prematurity or low birth weight				
No	Ref		Ref	
Yes	1.278 (0.640 to 2.554)	0.487	0.993 (0.438 to 2.253)	0.987
Pet owner				
No	Ref		Ref	
Yes	1.868 (0.741-4.712)	0.185	1.946 (0.684-5.540)	0.212
Remodeling history				
No	Ref		Ref	
Yes	1.092 (0.489-2.437)	0.830	1.074 (0.470-2.452)	0.866
Asthma				
No	Ref		Ref	
Yes	**5.914 (3.133 to 11.161)**	**<0.001**	**4.116 (1.913 to 8.857)**	**<0.001**
Allergic rhinitis				
No	Ref		Ref	
Yes	**1.974 (1.163 to 3.353)**	**0.012**	1.615 (0.904 to 2.886)	0.105
Atopic dermatitis				
No	Ref		Ref	
Yes	1.610 (0.957 to 2.709)	0.073	1.248 (0.691 to 2.254)	0.463

**(b):** 

Environmental factors	Univariated		Multivariated	
	Crude OR (95% CI)	*P* value	aOR (95% CI)^∗∗^	*P* value
Exposure to current smoking				
No	Ref		Ref	
Yes	**3.159 (1.360-7.335)**	**0.007**	**4.442 (1.831-10.776)**	**0.001**
Exposure to mold				
No	Ref		Ref	
Yes	**1.818 (1.098-3.009)**	**0.020**	**1.988 (1.168-3.383)**	**0.011**

aOR: adjusted odds ratio; CI: confidence interval; LBW: low birth weight. ^∗^Adjusted for gender, school grade, obesity, sensitization to aeroallergen, prematurity, and/or low birth weight. ^∗∗^Adjusted for gender, school grade, sensitization to pollen, and current asthma. Numbers in bold indicate significant differences (*P* < 0.05).

## Data Availability

The datasets used and/or analyzed during the current study are available from the corresponding author on reasonable request for clinical research purposes.
